# Optimizing MR-Guided Radiotherapy for Breast Cancer Patients

**DOI:** 10.3389/fonc.2020.01107

**Published:** 2020-07-28

**Authors:** Maureen L. Groot Koerkamp, Jeanine E. Vasmel, Nicola S. Russell, Simona F. Shaitelman, Carmel N. Anandadas, Adam Currey, Danny Vesprini, Brian M. Keller, Chiara De-Colle, Kathy Han, Lior Z. Braunstein, Faisal Mahmood, Ebbe L. Lorenzen, Marielle E. P. Philippens, Helena M. Verkooijen, Jan J. W. Lagendijk, Antonetta C. Houweling, H. J. G. Desiree van den Bongard, Anna M. Kirby

**Affiliations:** ^1^Department of Radiation Oncology, University Medical Center Utrecht, Utrecht, Netherlands; ^2^Department of Radiotherapy, The Netherlands Cancer Institute–Antoni van Leeuwenhoek Hospital, Amsterdam, Netherlands; ^3^Department of Radiation Oncology, University of Texas MD Anderson Cancer Center, Houston, TX, United States; ^4^Department of Clinical Oncology, The Christie NHS Foundation Trust, Manchester, United Kingdom; ^5^Department of Radiation Oncology, Medical College of Wisconsin, Milwaukee, WI, United States; ^6^Department of Radiation Oncology, Sunnybrook Health Sciences Centre, Toronto, ON, Canada; ^7^Department of Radiation Oncology, University Hospital and Medical Faculty, Eberhard Karls University Tübingen, Tübingen, Germany; ^8^Department of Radiation Oncology, Princess Margaret Cancer Centre, University Health Network, University of Toronto, Toronto, ON, Canada; ^9^Department of Radiation Oncology, Memorial Sloan Kettering Cancer Center, New York, NY, United States; ^10^Department of Oncology, Odense University Hospital, Odense, Denmark; ^11^Research Unit for Oncology, Department of Clinical Research, University of Southern Denmark, Odense, Denmark; ^12^Imaging Division, University Medical Center Utrecht, Utrecht, Netherlands; ^13^Department of Radiotherapy, Royal Marsden NHS Foundation Trust and Institute of Cancer Research, Sutton, United Kingdom

**Keywords:** breast cancer, neoadjuvant radiation therapy, partial breast irradiation, MR-guided radiotherapy, hybrid machine, MR-linac, magnetic resonance imaging (MRI)

## Abstract

Current research in radiotherapy (RT) for breast cancer is evaluating neoadjuvant as opposed to adjuvant partial breast irradiation (PBI) with the aim of reducing the volume of breast tissue irradiated and therefore the risk of late treatment-related toxicity. The development of magnetic resonance (MR)–guided RT, including dedicated MR-guided RT systems [hybrid machines combining an MR scanner with a linear accelerator (MR-linac) or ^60^Co sources], could potentially reduce the irradiated volume even further by improving tumour visibility before and during each RT treatment. In this position paper, we discuss MR guidance in relation to each step of the breast RT planning and treatment pathway, focusing on the application of MR-guided RT to neoadjuvant PBI.

## Introduction

The combination of a worldwide rising incidence of breast cancer together with decreasing mortality following breast cancer treatment has resulted in increasing numbers of breast cancer survivors living with late treatment-related toxicity (1–3). In recent decades, this has led to prioritization of treatment de-escalation aiming to reduce treatment-related toxicity without impeding survival (4). Studies comparing adjuvant whole breast irradiation (WBI) vs. adjuvant partial breast irradiation (PBI) in women with lower-risk breast cancers have demonstrated that PBI is as effective as WBI in terms of 5-years local recurrence rates and survival but with lower rates of late patient-reported and clinician-reported toxicity (5–8). Nonetheless, late treatment-related toxicity remains an issue in a significant proportion of patients (6, 8).

With neoadjuvant PBI, smaller target volumes can be irradiated compared to conventional adjuvant PBI, potentially resulting in less radiotherapy (RT)–related toxicity and therefore a higher quality of life (9–11). This is because, for neoadjuvant PBI, the gross target volume (GTV) is tumour rather than tumour bed, presenting a smaller, more easily definable target. Furthermore, the breast tissue at risk of local relapse remains in the closest possible proximity to the GTV, thereby reducing uncertainty around location of the clinical target volume (CTV). This is increasingly important in the current era of oncoplastic surgery in which the tissue that was adjacent to the tumour, the edge of which is usually marked by titanium surgical clips, may be mobilized and placed at some distance from its original location in order to ensure a good cosmetic result. This can lead to a larger CTV in the adjuvant setting than would have been necessary in the neoadjuvant setting. One problem with irradiating tumours in the neoadjuvant setting using the current standard computed tomography (CT)–based RT planning pathway, however, is that primary breast cancers can be difficult to see on a standard non–contrast-enhanced RT planning CT scan.

The development of magnetic resonance (MR)–guided RT has greatly improved the possibilities for image-guided RT and greater sparing of healthy tissue by providing excellent soft tissue visualization. MR–guided RT can refer to treatment on a conventional linear accelerator (linac) with the use of additional imaging on an MR scanner to plan treatment or to treatment on a hybrid machine. A hybrid machine is an MR scanner combined with a linac (MR-linac, Unity Elekta and MRIdian linac, ViewRay) or with ^60^Co sources (MRIdian, ViewRay) (12–15). For breast cancer patients, MR-guided RT is expected to be most beneficial in the neoadjuvant setting treating *in situ* tumours, which can be more clearly visualized on MR images than on CT, both at the time of RT planning and during RT treatment. The latter would facilitate reduction in setup error margins in both the neoadjuvant and adjuvant setting. In addition, administering MR-guided RT on a hybrid machine could reduce the radiation exposure associated with the daily cone-beam CT (CBCT) required during treatment on a conventional linac.

In this position paper, we discuss MR guidance in relation to each step of the breast RT planning and treatment pathway from simulation to contouring, to treatment planning, and then delivery. We review what is already known, what is under evaluation, and potential obstacles to clinical implementation, highlighting where optimization of techniques and/or workflow is still required (Table 1).

**Table 1:** Overview of challenges for the implementation of MR-guided radiotherapy on a hybrid machine for breast cancer patients.

**Challenge**	**Effect**	**Potential solution**
**SIMULATION**		
Patient positioning inside the MR bore	Prone: breast deformation on tableramya and fitting of receiver coil (Figure 2)	Development of a thinner coil or a dedicated MR-linac breast coil
	Supine: difficulties fitting arms inside bore in standard RT position	Use a minimal or no inclined wedge support, move arms closer together above the head
Deformation of body contour by receiver coil	Disturbed body contour	Use coil bridges to support the coil (Figure 1)
Body contour visibility in prone position	With dedicated prone breast coil, body contour and OARs not visible further away from coil	Use an additional coil placed on top of the patient
Electron stream effect	Irradiation dose outside the treatment field in an inferior-to-superior direction (Figure 4)	Include chin, arm, and abdominal region in the simulation plan
Breathing and cardiac motion during scanning	Motion artefacts	Use a 3D sequence, signal averaging, and left–right phase encoding in protocol design, or use triggering or breath-hold for acquisition
**CONTOURING**		
Surgical clip and/or marker visualization on MRI	Magnetic field distortion and artefacts impeding contouring of target volume (Figure 3)	1. Use or develop markers or clips with smaller artefacts 2. No marker insertion (only possible in the neoadjuvant setting if no further surgery is required)
**SIMULATION AND PLANNING**		
Geometric accuracy (gradient nonlinearities) in combination with lateral target volumes	Reduced geometric accuracy, increasing with distance from isocenter	1. Use distortion correction software on scanner 2. Position target as close to scanner isocenter as possible (e.g., shift patient on the table) 3. Include remaining inaccuracy in PTV margin
Geometric accuracy (magnetic field inhomogeneities and patient-induced distortions)	Reduced geometric accuracy, especially near tissue–air interfaces	1. Use high bandwidth acquisition 2. Acquisition of B0 map to assess patient-induced distortion.
**PLANNING**		
Electron return effect	Possible skin dose, chest wall, or lung dose increase (dose increase at tissue–air interfaces)	Pay attention to skin, chest wall, and lung dose constraints in planning, carefully choose beam setup (e.g., use enough beams)
Electron stream effect	Irradiation dose outside the treatment field in an inferior-to-superior direction (Figure 4)	Use of bolus material to shield irradiation outside of field
Missing electron density information in MR-only workflow	Inaccurate dose calculation without correct electron density assignments	Development of methods for synthetic CT generation from MRI
High-density treatment couch material	Unpredictable dose effects by daily replanning	Avoid beam angles passing through the treatment couch edges
**TREATMENT**		
Irradiation through coil	No irradiation through MR receiver coils, only through dedicated hybrid machine coils. Dedicated prone breast coil cannot be used	1. Try to fit the dedicated MR-linac coil on top of prone patient (only for smaller patients) 2. Design a thinner, more flexible coil for the hybrid system 3. Design a new prone coil for the hybrid system
Fixed treatment couch	Interfractional changes in position cannot be corrected by moving the treatment couch	Use online plan adaptation strategies to account for interfractional changes in anatomy
Motion during treatment	Geographical miss during treatment or increased PTV margins	Use online gating or tracking when available, e.g., only beam-on when the target volume is within pre-specified boundaries

*MRI, magnetic resonance imaging; MR, magnetic resonance; OAR, organ at risk; PTV, planning target volume; CT, computed tomography; RT, radiotherapy*.

## Simulation

### Patient Setup

The main challenge for patient setup in treatment position for breast RT in a magnetic resonance imaging (MRI) scanner or a hybrid machine is the limited MRI bore size (60–70 cm) compared to the CT bore size of 80 to 90 cm (16–18). This limits the size and inclination of a positioning device, as well as the number of possible positions for patient setup.

**Figure 1:**
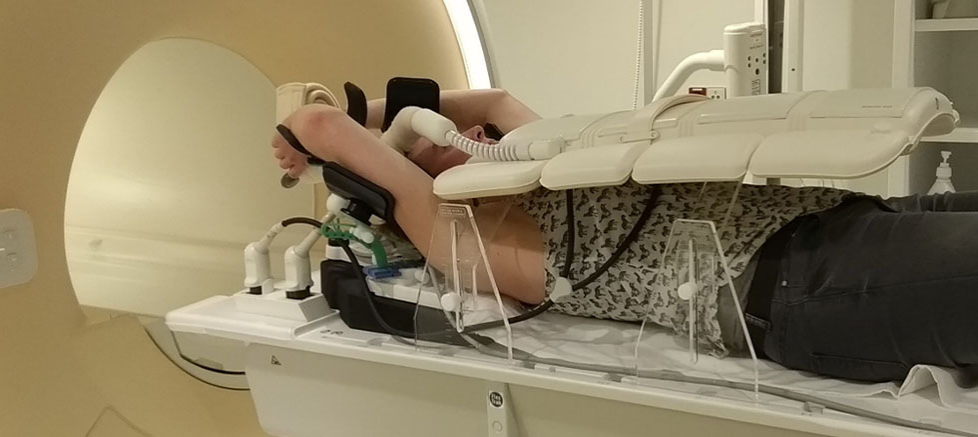
Supine patient setup for MRI simulation. In this setup, a 5-degree inclined wedge is used. Height-adjustable coil bridges are used as support for the anterior receiver coil to prevent deformation of the body contour.

For patients treated in supine position with arms raised above their head, the elbow span in combination with an inclined position can be problematic. A solution for this is to put the arms closer together and/or to use either a wedge with smaller inclination or no wedge at all. Placement of an anterior receiver coil on a patient in supine position could lead to deformation of the breast (19). However, coil bridges can be used as support for the coil to prevent deformation (Figure 1) (9, 20–22).

**Figure 2:**
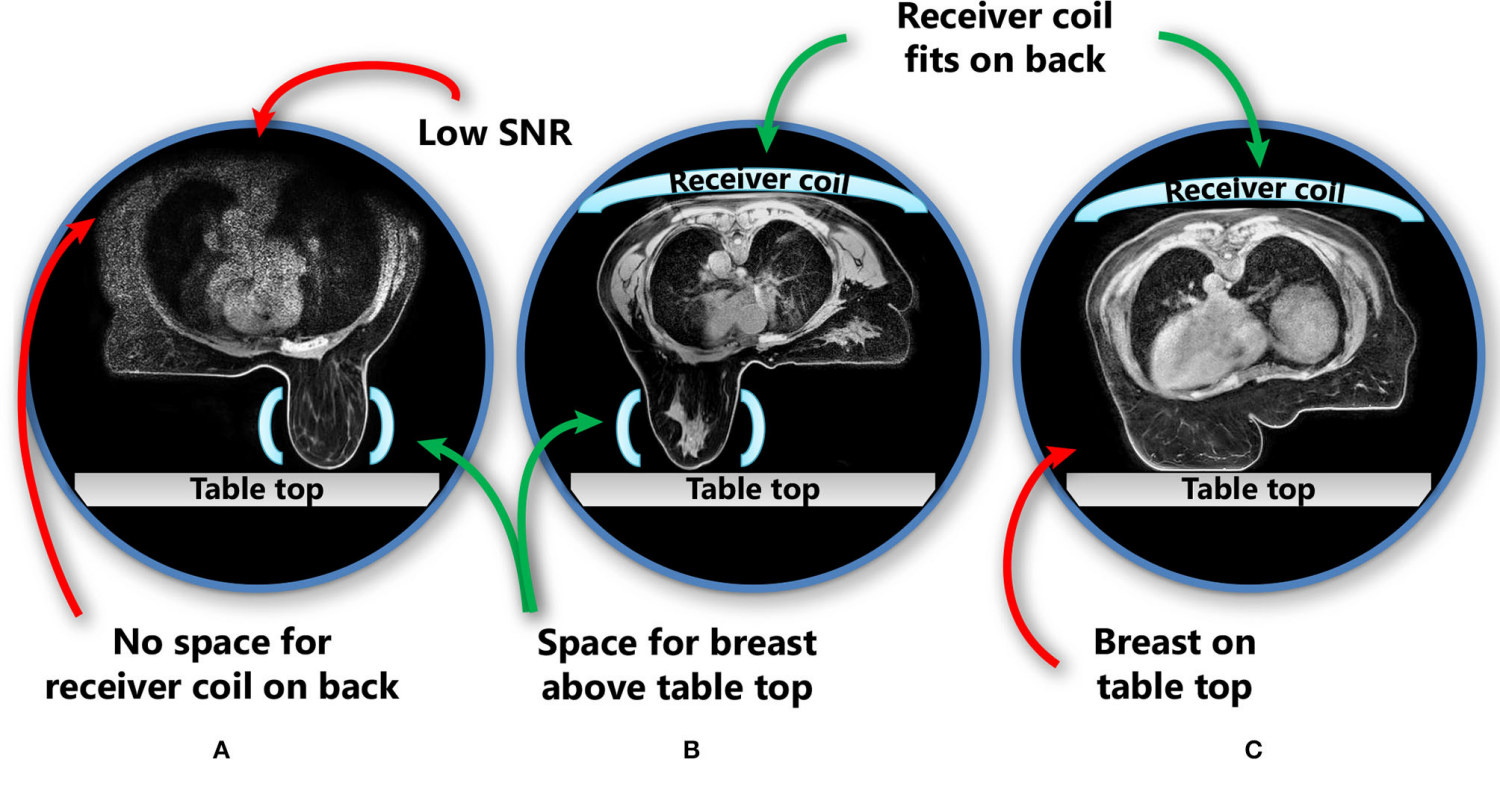
Patient and receiver coil positioning in prone position, including challenges in this position. The images show three different patients. **(A)** No space for the receiver coil on the back of the patient if the breast hangs freely without touching the scanner table; **(B)** the receiver coil fits above the patient while also the breast hangs freely; **(C)** when the receiver coil is fitted in the MRI bore above the patient, the breast touches the table top and is deformed. Light blue shapes represent the receiver coils (horizontal: receiver coil array; vertical: single flex coil). SNR, signal-to-noise ratio.

In the prone position, the proportion of patients who can fit into the MR scanner bore is limited by the space needed for a pendulous breast to hang freely without touching the table top in combination with the requirement to place an additional receiver coil on the back of the patient (Figure 2). The additional receiver coil is necessary as the full body contour is needed for RT planning purposes, which is not a requirement for diagnostic prone breast imaging.

Standard RT immobilization equipment may not necessarily be MR-compatible, and standard MR equipment (e.g., the dedicated prone breast coil) is not designed for setup reproducibility. Therefore, it is necessary to develop dedicated RT immobilization equipment that is MR-compatible (i.e., non-conductive, low-density material). This equipment must also fit inside the MR bore and leave room for the MR receiver coils (e.g., flexible receiver coils in a prone breast board), while not degrading image quality (17, 22). Because of the electron stream effect (ESE), further discussed in *Treatment Planning for a Hybrid Machine*, simulation should include the chin and upper abdominal region.

### Image Quality

For optimal quality of MR images, the receiver coil should be placed close to the target volume. Therefore, a strategic setup for the additional coils should be chosen, specific to the selected patient position (e.g., supine or prone). Because RT immobilization devices, such as the supine and prone breast boards and coil bridges, increase the gap between the patient and the receiver coils (i.e., the distance to the posterior coil located in the scanner table and to the anterior coil on top of the patient), it was initially thought that the positioning requirements for breast cancer RT might have a negative impact on MR image quality. However, multiple studies have reported good quality of MR images for breast RT in both supine and prone treatment positions acquired at 1.5- and 3.0-T MR scanners (19, 21, 22).

Another factor that might impair MR image quality is organ motion, including respiratory and cardiac motion, during scanning. Imaging in prone position has the advantage of minimizing breast motion due to respiration and may also minimize motion artefacts (19). Batumalai et al. (22) found no significant effect of the breathing artefacts on image quality in both prone and supine position by instructing their volunteers to maintain shallow breathing and choosing a right–left phase encoding direction in their MRI scans. Additionally, to preventing the motion, artefact reduction (e.g., gating or triggering) or motion correction (e.g., MR navigators) techniques can be used to minimize motion effects on MRI scans. However, it is important to realize how the anatomy relates to the breathing state during RT (18). To prevent step-like displacements in different slices in the scan volume caused by motion during scanning, a three-dimensional (3D) sequence can be used, although motion in a 3D scan will lead to blurring (23).

In studies that evaluated prone breast MRI for RT, a dedicated breast coil is usually used (19, 22, 24). While this coil provides optimal image quality for the breasts, it cannot capture the full body contour and all organs at risk (OARs) with adequate quality (Figure 2). However, for MR-guided RT on a conventional linac, this may be sufficient, provided that enough anatomical landmarks are visible to register the MR scan to the planning CT scan. Scanning with an additional receiver coil on top of the patient could help to overcome this issue, but may not be possible in all patients because of the limited MR bore size.

In case of RT treatment on a hybrid MR-guided RT system, it is not possible to irradiate through the standard dedicated prone breast coils that are used in diagnostic MRI. For that reason, the receiver coils dedicated to hybrid machines have a “window” through which irradiation is possible (15, 25). Because these dedicated coils have different properties to the standard receiver coils (i.e., fewer coil arrays, which restricts acceleration of imaging) and are not breast specific, the image quality can be inferior. Another restriction is that the coil cannot be placed too closely to the patient because of the electron return effect (ERE; see *Treatment Planning for a Hybrid Machine*), which restricts the signal-to-noise ratio of the imaging. In general, a higher field strength gives a better signal-to-noise ratio, which may place a 1.5-T hybrid system in favour over a 0.35-T system. However, experiences with the 0.35-T hybrid system show that patient setup and online tracking for breast cancer could be performed successfully based on imaging at this lower magnetic field strength (26). To ensure appropriate image quality, the MRI sequences and image quality for breast imaging on the hybrid systems should therefore be tested and optimized for the use of the dedicated coil and each system specifically.

### Geometric Accuracy

The impact of geometric distortions on MR-based contouring and planning should be taken into account when optimizing image quality and MRI sequences for RT on a hybrid machine (18, 27). The effect of distortions on image quality is described in this section, whereas the effect of distortions on dose distributions is described in *Treatment Planning for a Hybrid Machine*.

Distortions arise from system-related factors (i.e., main magnetic field inhomogeneity and gradient nonlinearities) and patient-related factors (i.e., chemical shift and susceptibility effects) and depend on the specific scanner and sequence parameters (18, 28–31).

System-related distortions due to gradient non-linearities increase with increasing distance of the target volume from the MRI isocenter and can range up to 12 mm (25, 27, 28, 30, 32). For the Elekta MR-linac (1.5 T), maximum displacements of 2.0 mm were found within 17.5 cm from the isocenter (25). For the ViewRay ^60^Co-system (0.35 T), this was 1.9 mm, but larger distortions were observed further from the central axis (33). To minimize the effect of image distortion by gradient non-linearities, the target volume should be positioned as close to the scanner isocenter as possible (17), which may be challenging for laterally located target volumes, such as lateral breast tumours. A possible solution may be to shift the patient on the scanner table toward the contralateral side such that the ipsilateral breast moves closer to the machine isocenter, if this is possible within the limited space inside the bore. Furthermore, to minimize system-related distortions, it is also important to always use the scanner's software for gradient non-linearity correction (23, 30). By using a 3D scan, the gradient non-linearity correction can be applied in all directions.

Distortions caused by main magnetic field inhomogeneities and by susceptibility effects induced by the patient's presence in the scanner also need to be corrected for. Distortion caused by patient-induced susceptibility can be particularly large, especially at the tissue–air interface, with mean maximum distortions at 3.0 T having been found to increase from 1.4 to 3.7 mm in a phantom to 3.7 to 11.3 mm in patients (including setup uncertainties) (29). Susceptibility effects scale with the main magnetic field strength (31). A lower field strength or a high receiver bandwidth can help to reduce both main magnetic field inhomogeneity and patient-induced susceptibility, but reduces signal-to-noise ratio (30, 31). Patient-specific correction methods (e.g., using the B0 map) may be helpful to correct for these distortions (18, 30).

### Choice of MR Image Contrast

Several MRI sequences have been recommended for MR-guided RT. For use of MRI in the adjuvant breast RT setting, use of T1-weighted 3D sequences without fat suppression resulted in the best visualization of surgical clips, whereas T1-weighted images with fat suppression (e.g., mDixon) best enabled differentiation between glandular breast tissue and seroma (9, 20, 34). Two-dimensional or 3D T2-weighted MRI with fat suppression [e.g., Short inversion time inversion recovery (STIR) or water selective excitation] or without fat suppression was preferred for visualization of lumpectomy cavity and associated seroma and for discrimination between glandular breast tissue and tumour bed (17, 19, 21, 22, 24).

**Figure 3:**
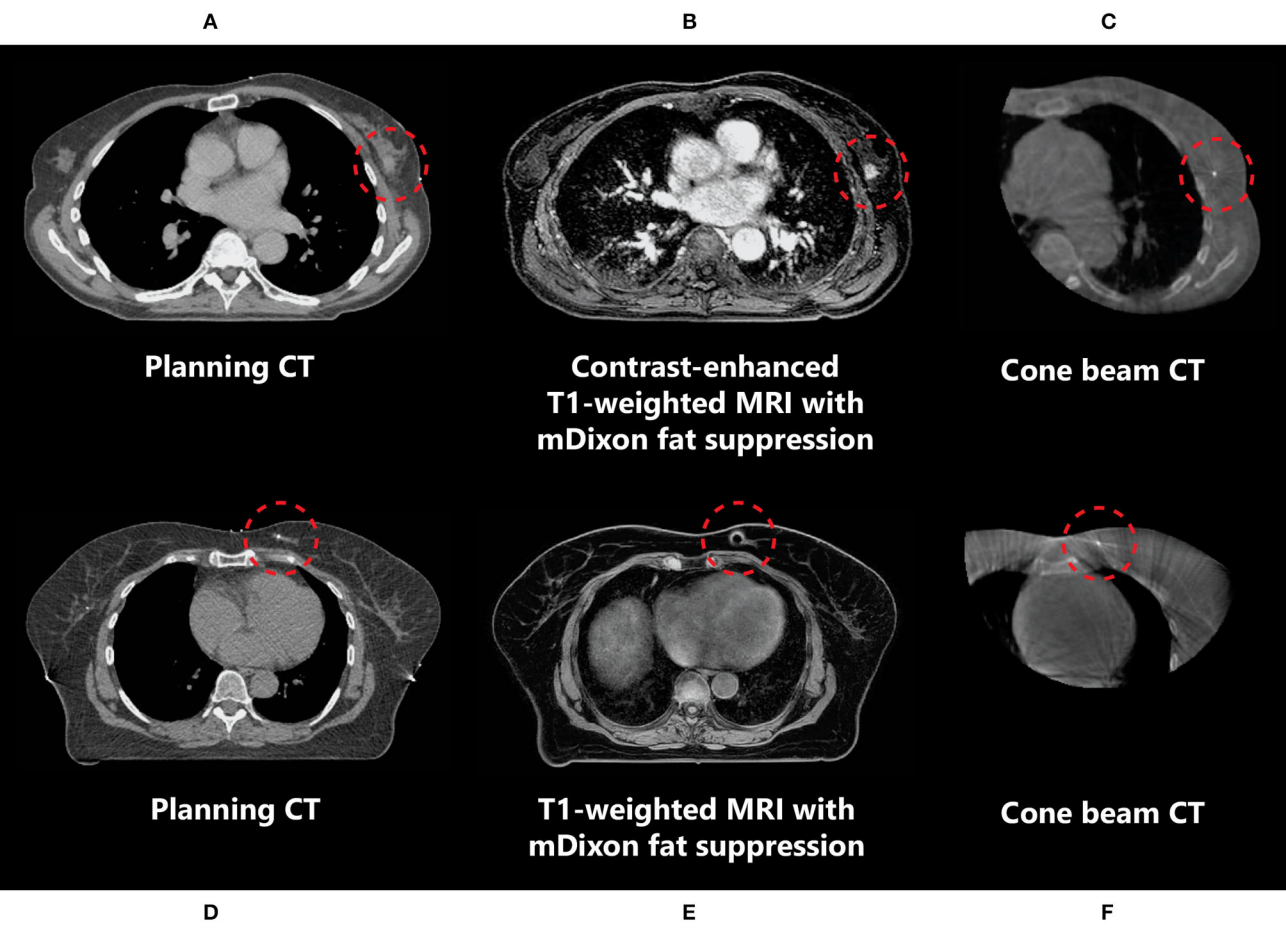
Imaging of a primary breast tumour on CT **(A,D)**, (contrast-enhanced) MRI **(B,E)**, and CBCT **(C,F)** scans indicating the difference in tumour visibility (inside the red circle) between these modalities in two different patients (**A–C** and **D–F**). **(D–F)** The marker inserted in the tumour medial in the left breast is observed as a void on MRI (indicated by the red circles).

In the neoadjuvant setting, the use of T1-weighted fat-suppressed contrast-enhanced MRI is recommended for optimal tumour and tumour spiculae visualization, because differences in contrast uptake provide a clear distinction between tumour and glandular breast tissue (Figure 3) (35–38). Additionally, T2-weighted images might aid in the differentiation between tumour and postbiopsy changes (35). mDixon fat suppression methods proved to be reliable and are recommended because they are relatively insensitive to main magnetic field inhomogeneities (39, 40). Use of diffusion-weighted imaging (DWI) was described in only one study, where it was used in the context of response evaluation after RT and not for target delineation (35). Use of DWI for RT could help in differentiation between benign and malignant lesions, but magnetic susceptibility-induced geometric distortions make it more suitable for diagnostic imaging than for MR-guided RT (41, 42). All studies presented above used fusion of MRI with a planning CT scan on which the OARs were delineated. Therefore, no recommendations focusing on OAR visualization on different MRI sequences have been published. Based on expert opinion, OARs are clearly visualized on any of the sequences mentioned above, except for DWI. All sequences described were acquired on stand-alone MRI scanners. Hybrid treatment machines may come with only a fixed set of available MRI sequences in clinical mode (15, 43). Therefore, not all sequences described may be available on these machines during treatment. A summary of online available MRI sequences on hybrid machines is presented in Table 2.

**Table 2:** Overview of recommended MR sequences and commercial online availability for clinical breast cancer treatment on hybrid machines.

**Type of MR sequence**	**Advantages (+) and disadvantages (-)**	**Availability on Unity (Elekta AB)**	**Availability on MRIdian^®^ (ViewRay^®^)**
**Postoperative**			
T1-weighted with fat suppression (9, 20, 34)	+ Differentiation between glandular breast tissue and seroma	Not available^*^	Not available
T1-weighted without fat suppression (9, 20, 34)	+ Best visualization of surgical clips	3D T1-weighted FFE	3D T2/T1-weighted TRUFI
T2-weighted with or without fat suppression (17, 19, 21, 22, 24)	+ Visualization of lumpectomy cavity and seromaramya + Differentiation between glandular breast tissue and seroma	3D T2-weighted TSE without fat suppression^*^	3D T2/T1-weighted TRUFI
DWI (35)	+ Differentiation between malignant and benign tissue in case of irradical resection ramya – Susceptible to geometric distortions	Not available^*^	Not available^*^
**Preoperative**			
T1-weighted contrast-enhanced with fat suppression (35–38)	+ Visualization of tumour and tumour spiculae ramya – Injection of and irradiation with contrast agent	No standard contrast injection available	No standard contrast injection available
T2-weighted with or without fat suppression (35)	+ Differentiation between tumour and post-biopsy changes	3D T2 TSE without fat suppression^*^	3D T2/T1-weighted TRUFI
DWI (35)	+ Differentiation between malignant and benign tissue ramya – Susceptible to geometric distortions	Not available^*^	Not available^*^

*TSE, turbo spin echo (fast spin echo); FFE, fast field echo (spoiled gradient echo); TRUFI, true fast imaging with steady state precession (balanced steady state free precession)*.

* *Not available in online treatment setting. Acquiring DWI and MR sequences with fat suppression is possible offline—outside online treatment setting mode*.

*This table does not provide an exhaustive overview of all imaging possibilities but only refers to MR sequences mentioned in this article and currently commercially available imaging options*.

## Contouring

With regard to target volume delineation in the adjuvant PBI setting, delineation of the tumour bed on CT should, according to guidelines, include visible seroma and representative surgical clips and the tumour location on preoperative imaging and take into account the microscopic tumour free margins (44–47). The added value of MRI to a standard planning CT scan for delineation in the adjuvant setting is disputed for several reasons (48, 49). First, surgical clips lead to voids on MRI, potentially leading to less accurate target volume definition (34). Second, studies have shown both a significant increase as well as a decrease in the target volume when either a preoperative or postoperative MRI scan was available for delineation in addition to a postoperative planning CT (20, 21, 34, 50). Third, in three separate studies, MRI did not lead to a reduction in interobserver variation (20, 24, 50). However, in a more recent larger study, a significant reduction in interobserver variation was reported for delineation on MRI in patients without surgical clips (51). Therefore, the added value of using MRI for contouring in the adjuvant setting seems likely to be limited to those patients in whom tumour bed clips have not been placed.

In the context of neoadjuvant PBI, given that this is not yet a standard of care in breast cancer management, delineation of *in situ* breast tumours is a relatively new concept to most radiation oncologists, and new guidelines are needed. Guidelines for the delineation of primary breast tumours on MRI for use in neoadjuvant PBI setting have recently been developed by the Breast Tumor Site Group of the International MR-Linac Atlantic Consortium (36). These recommend the use of contrast-enhanced MRI, which, because of increased contrast uptake in tumours compared to the surrounding glandular breast tissue, allows for better visualization of breast tumours than using CT (Figure 3) (9, 38). Contrast-enhanced MRI has been used for the delineation of target volumes in several recent studies of neoadjuvant PBI (37, 52). In these studies, insertion of an additional fiducial marker by a radiologist was necessary both to help localize the tumour for subsequent surgical resection in case of tumour downstaging and for tumour position verification because the tumour cannot be visualized on CBCT in most patients. These markers cause artefacts on MRI, which can be observed as voids (Figure 3). The size of these artefacts depends on the material and geometry of the marker. As the artefact can obscure tumour tissue, the void of a marker should be included in the target volume. If omission of surgery after an ablative dose RT becomes clinically feasible, insertion of a fiducial marker in the tumour might not be necessary anymore. This would be beneficial for both target volume definition and follow-up imaging, as well as patient satisfaction (53).

## Treatment Planning for a Hybrid Machine

For MR-guided RT on a conventional linac, treatment planning is performed according to the standard practice. This includes registering the MRI scan to the planning CT scan used for delineation and producing a dose distribution using a standard treatment planning system. However, when treatment is to be delivered on a MR-guided hybrid machine, several additional factors need to be considered, all of which will be incorporated into the dedicated treatment planning systems. These factors are inherently related to the design of the hybrid machines. First, given that the magnetic field influences the path of secondary electrons, the ERE and the ESE in air have to be taken into account. Second, the influence of geometric accuracy of the MR images on treatment planning must be considered. Third, there are some restrictions for planning to bear in mind.

### Electron Return Effect

The Lorentz force acting on moving charged particles in a magnetic field causes several effects during irradiation in a magnetic field (54–59). One of these is the ERE, which refers to the fact that the path of electrons is bent in the presence of a magnetic field, resulting in exit electrons re-entering the body after a helical path in air (55). Studies have shown that skin dose is increased for patients undergoing WBI in a magnetic field due to the ERE (60, 61). According to van Heijst et al. (60), the mean skin dose increased from 29.5 Gy at 0 T to 32.3 Gy at 0.35 T and to 33.2 Gy at 1.5 T for 2-beam WBI. For 7-beam WBI, the mean skin dose increased from 27.9 Gy at 0 T to 30.2 Gy at 0.35 T and to 29.8 Gy at 1.5 T. Given these findings, WBI is not thought to be a good indication for treatment on a hybrid machine, irrespective of the field strength. Although van Heijst et al. found that the mean skin dose for PBI also increased, from 5.2 Gy at 0 T to 5.6 Gy at 0.35 T and 5.8 Gy at 1.5 T, the absolute mean skin dose was small compared to WBI. Therefore, the increase in skin dose for PBI in a magnetic field would be highly unlikely to translate into a higher risk of radiation dermatitis. Furthermore, it has been reported that increasing the number of beam angles helps in decreasing the skin dose (60, 62). Therefore, although PBI is a good indication for breast RT on a hybrid machine, one should remain aware of the risk of increased skin dose and use more rather than fewer beams. Because the ERE effect is also present at the lung–tissue interface, it is also important to check the maximum lung and chest wall dose (57, 62). Previous planning studies concluded that the effects of the magnetic field on OARs, other than the skin, are generally negligible, and doses were within clinical constraints (60, 62, 63).

### Electron Stream Effect

**Figure 4:**
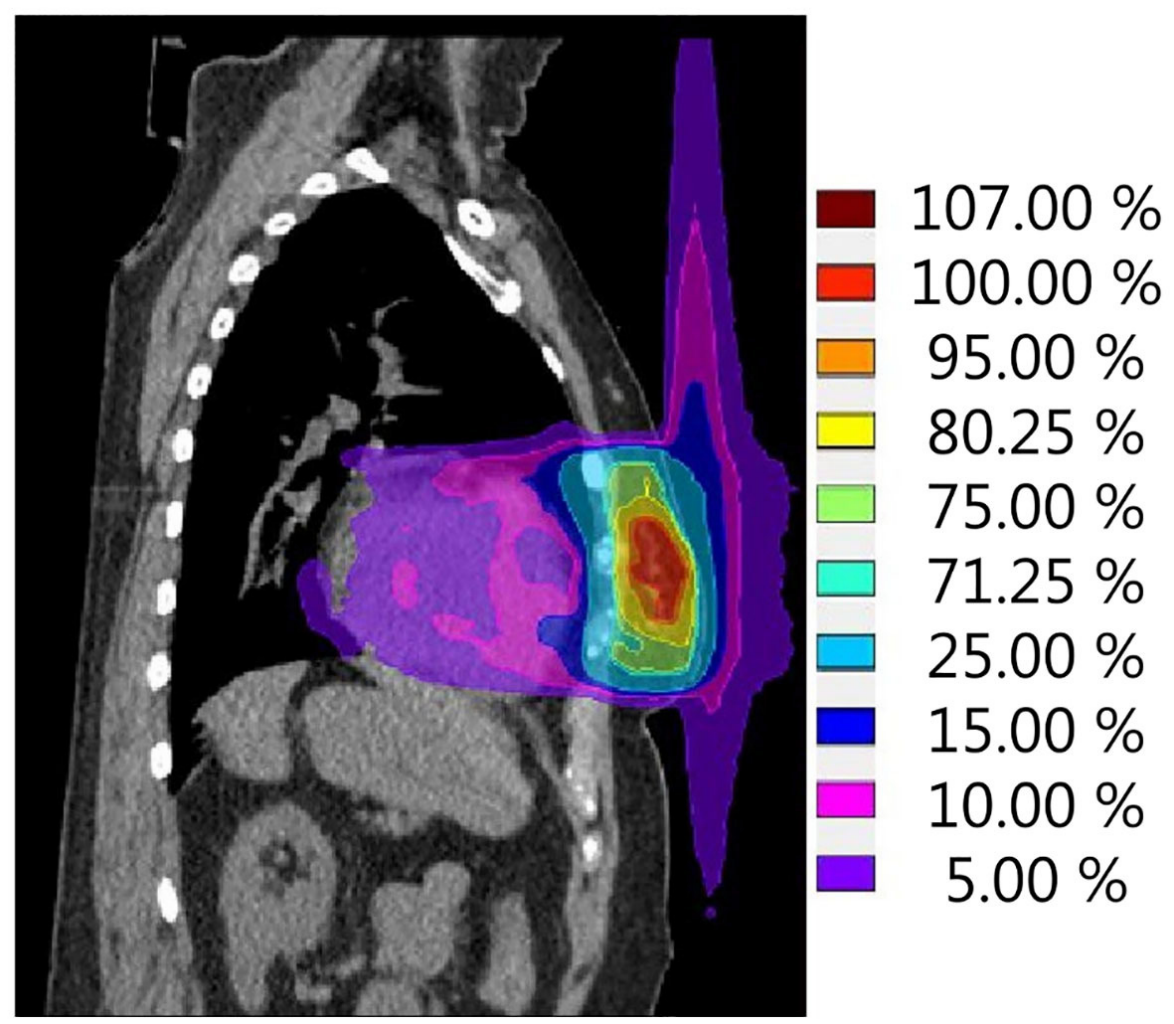
Simulation of a single fraction neoadjuvant PBI treatment plan (ABLATIVE trial approach, 1 × 20 Gy to GTV) for the 1.5-T MR-linac. The calculated dose distribution shows the electron stream effect in air resulting in dose outside of the treatment field in both cranial and caudal directions. Scale is set to 100% reference dose = 20 Gy.

The second effect that should be kept in mind for breast cancer treatment on a hybrid machine is the ESE in air, which can lead to dose being deposited in tissues well outside the irradiated field (Figure 4). This was first observed and evaluated by Park et al. (64), who, in the context of accelerated PBI delivered on the 0.35 T ^60^Co ViewRay system, observed an electron stream in air extending toward the head and ipsilateral arm. This ESE is caused by electrons generated inside the body that, instead of scattering in random directions when leaving the body, start spiraling along the magnetic field (65). If unobstructed, this electron stream would reach the chin and arm, causing unwanted irradiation of the skin in these areas. In an extreme case, the maximum dose measured was as high as 16.1% of the prescribed dose (64). Dose to the skin outside the treatment field was highest in patients with tumours located in the cranial part of the breast. Depending on the location of the high-dose region in the breast, this electron stream can also be directed toward the feet (Figure 4). Studies on phantoms and early clinical experiences suggest that the treatment planning system is able to fully describe the ESE and that the use of bolus material to shield the body parts located in the electron stream showed effective reduction of the dose in these regions (64–66).

### Impact of Geometric Distortions

Because the breast is located peripherally in the body and geometric distortions increase with distance from the isocenter and susceptibility effects arise near tissue–air interfaces (as described in *Simulation*), the effects of these distortions on dosimetry for breast RT may be significant (27, 30). The system-specific distortions together with patient-related distortions may result in unacceptable dosimetric variations, as has already been shown for WBI (29). This issue still requires investigation in the context of PBI, such as investigation of the impact of distortion at the edges of the breasts, which would lead to inaccurate assignment of air vs. tissue electron density and therefore inaccurate dose calculations when these are based on the MRI. Geometric distortions inside the target region should be carefully considered in choosing adequate planning target volume (PTV) margins in the context of breast RT on an hybrid machine (33).

### Planning Restrictions

Technical specifications such as the magnetic field strength, beam energy, source-to-axis distance, and maximum field size are system-specific and are accounted for in the treatment planning systems (13–15). However, there are some specific issues to highlight that will be different from treatment planning for breast irradiation on a conventional linac. First, for the ViewRay MR-linac system angles between 30 and 33° are not available, whereas for the Elekta system 8 to 18° degrees need to be avoided because of the cryostat pipe (15, 67). Furthermore, some beam angles commonly used for breast RT on conventional systems should preferably not be used on the Elekta system, that is, angles around 130–150° and 210–230°, with exact angles depending on the tumour location (66, 67). This is because of high-density material in the treatment couch edges that may cause unwanted dose effects during daily plan adaptation. Because of the design of the hybrid machines, rotations of the table with respect to the gantry angle and therefore irradiation with non-coplanar beams are not possible. No problems are expected because of this because good plan quality for PBI can be achieved with coplanar IMRT (26, 63, 68).

With respect to the methods currently used for dose calculation, co-registration of the planning CT to the pre-treatment and/or online MR images or bulk density assignment are currently used for electron density information for both the ViewRay and Elekta hybrid machines (15, 67, 69). Strategies for creating a synthetic CT directly from an MRI scan, such as atlas-based, voxel intensity–based, or deep learning approaches, are in development (70, 71). However, data on the use of synthetic CT for the breast or thoracic region are limited. Recent data have shown encouraging results for synthetic CT generation for the thoracic region based on (a combination of) voxel intensity– and atlas-based approaches, with a mean absolute error <50 HU in the body and dosimetric differences ≤ 1.7% inside lung tumour PTVs (72, 73). Inclusion of bone density information, specifically the spine in this study on lung tumour treatment plans, proved to be important to reduce local hot spots in the differences between the simulated dose distributions on CT and synthetic CT (73). Ahunbay et al. (74) proposed to continue using a planning CT scan for each patient. Their approach with inclusion of bone density and the use of deformably registered lung density, both of which may be necessary for breast RT treatment planning as well, may enable accurate full online replanning on the daily anatomy. In an online workflow, options may be limited by the specific system, but aforementioned issues should be taken into account, as well as speed of synthetic CT generation.

## Treatment on a Hybrid Machine

For MR-guided RT on a conventional linac, the treatment and position verification can be performed according to the current standard RT workflow. Using a hybrid machine with daily online MRI both before and during treatment, new opportunities become available for daily setup and positioning accuracy, online adaptive RT based on daily anatomy, and intrafraction motion management.

### Daily Setup and Positioning Accuracy

Experiences from hospitals that have treated breast cancer patients in the adjuvant setting with the 0.35 T ^60^Co system have shown that initial patient setup verification based on location of lumpectomy cavity and online motion monitoring could be beneficial for PBI patients in terms of reducing the CTV to PTV margin and therefore irradiated volume and thereby the risk of late toxicity (26, 43, 69, 75). A >52% reduction in treatment volume was achieved by applying no PTV margin for the lumpectomy cavity with the help of online MRI for setup (26, 75). Although a 0-mm PTV margin neglects correction of other uncertainties that would normally be incorporated in the CTV to PTV margin (e.g., mechanical equipment and dosimetric uncertainties) (76), this illustrates that online MRI for setup may help to reduce the PTV margin compared to treatment on a conventional linac. With the aid of an online motion monitoring approach, a mean difference of <1% between planned and delivered dose to 95% of the target volume was achieved (26). For treatment in the neoadjuvant setting, patient setup and positioning accuracy on a hybrid machine are still to be evaluated.

### Online Adaptive RT

On hybrid machines, a new treatment plan can be made during each fraction based on online MRI. Depending on the specific system, different strategies are available. These range from dose recalculation on the new patient anatomy to full online recontouring and replanning (15, 77, 78). Requirements for online replanning are somewhat different than for pretreatment planning. In particular, the time available for target and OAR redelineation and plan optimization is much reduced because the patient is on the treatment table. The choice of plan adaptation strategy will therefore depend on a trade-off between plan quality and speed of plan adaptation. In general, it is expected that a full reoptimization plan adaptation method will lead to improved dosimetry in most patients, especially in the case of deformations in the tumour or OARs, but will take more time (78, 79). In the group reporting on adjuvant PBI on a ^60^Co system, where online MRI proved beneficial for setup and PTV margin reduction, no online plan adaptation was performed, and yet retrospective comparison of planned vs. delivered dose showed adequate coverage, suggesting that, in the context of PBI, use of a simpler plan adaptation strategy may be reasonable (26, 43). Currently, injection of contrast agent is not performed during treatment on a hybrid machine, although it could help to recontour the tumour volume in case of neoadjuvant PBI. However, gadolinium chelates, the most commonly used contrast agent for breast cancer, could have a radiosensitizing effect (80). Because of the uncertainty of the effect and safety of irradiation when a contrast agent has been injected and concern about stability and toxicity of irradiated gadolinium, it is not recommended to use contrast-enhanced sequences for imaging during treatment.

### Intrafraction Motion Management

Generally three types of intrafraction motion can be distinguished: (1) regular breathing motion, (2) irregular transient motion, and (3) non-transient bulk motion. Breast intrafraction motion evaluated on 2D and 3D MR images (2- to 20-minutes duration) has been reported to be generally regular and limited to <3 mm (26, 81). Larger displacements have been observed, but these were mostly transient. Acharya et al. (26) calculated that a mean PTV margin of 0.7 mm would be sufficient to cover 90% of the lumpectomy cavity for 90% of the treatment time for a mean fraction duration of 12.7 minutes. However, intrafraction displacement seemed to differ substantially between patients, reaching a mean displacement range of 6 mm in anterior-posterior direction for one patient. One possibility to handle intrafraction displacement might be to individualize the PTV margin based on cine MR data from simulation. Larger whole-body shifts of up to 14 mm over a 21-minutes duration have been observed infrequently, although for the majority of patients motion evaluated up to 20 minutes was generally regular and small (81). The impact of intrafraction motion on current standard hypofractionated treatment is therefore likely to be limited. However, for extremely hypofractionated treatment schedules (one to two fractions) delivered on hybrid machines, treatment times will increase significantly because of the online delineation and planning procedure and because of increased beam on time because of a lower dose rate of the hybrid machines and use of IMRT compared to volumetric modulated arc therapy (68, 82, 83). This will increase the risk of systematic non-transient patient displacement both before and during treatment and may also negatively affect patient comfort. Although not yet available, real-time plan adaptation during RT delivery will be the ultimate goal to account for intrafraction motion management (84). Henke et al. (43) noted that online motion tracking and gating on the lumpectomy cavity were beneficial for accelerated PBI treatment with regard to reduction of the PTV margin (26, 43). A disadvantage of gating is that, although it is a solution for intrafraction motion management, it will even further increase the treatment time. Solutions for online monitoring and management of intrafraction motion such as cine MRI-based gated irradiation are not yet implemented for the 1.5-T Elekta MR-linac.

### First Clinical Experiences

Several publications have reported on neoadjuvant MR-guided PBI on a conventional linac including favourable toxicity profiles (35, 37, 85). However, no patients have yet been treated with neoadjuvant PBI on a hybrid machine. A planning study has shown that neoadjuvant PBI in a single fraction in prone or supine position on the 1.5-T Elekta MR-linac would be dosimetrically feasible with adequate target coverage and within predefined constraints for OAR (63).

Experiences with adjuvant PBI on a hybrid system have been published. For patients treated on the 0.35-T ^60^Co Viewray system with single-fraction adjuvant PBI, up to 12 months' follow-up is available, and no local recurrences have been reported. The first clinical results showed good tolerability, low toxicity with a maximum of grade 2 toxicity, and good to excellent cosmetic outcome assessed by both patients and physician (86, 87). Usage of this system resulted in benefits for initial patient setup on lumpectomy cavity and online motion monitoring by which the PTV margin was diminished to 0 mm, which led to a large reduction in treatment volume of 52% (26, 43, 69, 75). The first patient has also been successfully treated with adjuvant PBI in 15 fractions on a 1.5-T Elekta MR-linac, which led to only grade 1 toxicity of the breast with adequate protection of the chin to prevent unwanted irradiation due to the ESE (66).

Patients are currently being recruited for several studies on MR-guided PBI. On ClinicalTrials.gov, two trials are registered aiming to treat patients in the adjuvant setting on a hybrid machine, looking primarily at either reproducibility of treatment or cosmetic outcome (88, 89). Three other trials are being conducted to further explore the effect of neoadjuvant MR-guided PBI on a conventional linac (90–92). The primary outcomes of these trials are postoperative complication rate, reproducibility of treatment, and pathologic response, respectively.

## Conclusion

The addition of MR guidance to the breast RT planning pathway facilitates target volume delineation in the neoadjuvant PBI setting, whereas treatment on a hybrid MR and linac or ^60^Co machine could lead to reduced CTV to PTV margins in the neoadjuvant and adjuvant PBI settings through clearer visualization of the target volume during treatment. Although challenges for treatment of breast cancer patients on these systems remain (Table 1), the first breast cancer patients have been treated successfully with adjuvant PBI on a hybrid system, and studies of MR-guided neoadjuvant PBI will open shortly, through which technical approaches and workflow are likely to be further refined.

## Ethics Statement

Written informed consent was obtained from the patients and volunteers for the publication of any potentially identifiable images or data included in this article. Medical images presented in this work were from subjects participating in the ABLATIVE trial (ClinicalTrials.gov identifier: NCT02316561) and were used with permission from the ABLATIVE trial team. The ABLATIVE protocol was approved by the Institutional Review Board of the University Medical Center Utrecht, Utrecht, the Netherlands.

## Author Contributions

AK, HB, AH, MG, and JV contributed to conception and design of this work. MG and JV wrote the first draft of the manuscript. All authors contributed to data interpretation, critical manuscript revision, read, and approved the submitted version.

## Conflict of Interest

All authors are part of the Elekta MR-Linac Research Consortium. The aim of this consortium is to coordinate international research into the 1.5 T Elekta MR-linac. All authors have received financial support from Elekta for visiting consortium meetings. Elekta had no role in the preparation, review, or approval of the manuscript, and decision to submit the manuscript for publication.
